# The functional diversity of Aurora kinases: a comprehensive review

**DOI:** 10.1186/s13008-018-0040-6

**Published:** 2018-09-19

**Authors:** Estelle Willems, Matthias Dedobbeleer, Marina Digregorio, Arnaud Lombard, Paul Noel Lumapat, Bernard Rogister

**Affiliations:** 1 Laboratory of Nervous System Diseases and Therapy, GIGA-Neuroscience, University of Liège, Avenue Hippocrate, 15, 4000 Liège, Belgium; 2 Department of Neurosurgery, CHU of Liège, Liège, Belgium; 3 Department of Neurology, CHU of Liège, Liège, Belgium

**Keywords:** Aurora kinase, Mitosis, Cancer

## Abstract

Aurora kinases are serine/threonine kinases essential for the onset and progression of mitosis. Aurora members share a similar protein structure and kinase activity, but exhibit distinct cellular and subcellular localization. AurA favors the G2/M transition by promoting centrosome maturation and mitotic spindle assembly. AurB and AurC are chromosome-passenger complex proteins, crucial for chromosome binding to kinetochores and segregation of chromosomes. Cellular distribution of AurB is ubiquitous, while AurC expression is mainly restricted to meiotically-active germ cells. In human tumors, all Aurora kinase members play oncogenic roles related to their mitotic activity and promote cancer cell survival and proliferation. Furthermore, AurA plays tumor-promoting roles unrelated to mitosis, including tumor stemness, epithelial-to-mesenchymal transition and invasion. In this review, we aim to understand the functional interplay of Aurora kinases in various types of human cells, including tumor cells. The understanding of the functional diversity of Aurora kinases could help to evaluate their relevance as potential therapeutic targets in cancer.

## Mitosis

In physiological conditions, mitosis is induced by activation of the Cyclin B1-CDK1 complex, which controls the transition of the G2/M checkpoint. Mitotic entry also involves maturation, duplication and positioning of the centrosomes at the cell poles. During prophase, centrosome attachment to microtubules (MT) and cycles of MT (dis)assembly are required for the formation of the mitotic spindle. In pro-metaphase, the nuclear envelope breaks down (NEBD) and chromosomes start to condense and bind kinetochores. Chromosomes then align and adopt a bi-orientation during metaphase. In the meanwhile, the spindle assembly checkpoint controls kinetochore attachments to the MT.

Chromosomes then separate into two genetically identical sister chromatids, which move toward the spindle poles during anaphase A. During anaphase B, the spindle poles move apart to separate the two sets of sister chromatids. The movements of the sister chromatids and spindle poles are both mediated through kinetochore and MT dynamics. During telophase, nuclear membranes organize around each set of sister chromatids, which start to decondense. Finally, cytokinesis is induced by actin reorganization, leading to the formation of a contractile ring and a cleavage furrow. As a result, mitosis generates two daughter cells owning an equal number of chromosomes and cytoplasmic components as the parent cell [1].

## Mitotic kinome

Mitotic cells sometimes fail to equally distribute chromosomes to the daughter cells, leading to aneuploidy. Aneuploidy and chromosome instability can generate clones with tumor-promoting properties. Indeed, aneuploidy characterizes 90% of human tumors and aggravates tumor heterogeneity, drug resistance and recurrence [2]. Mitosis, therefore, needs to be rigorously regulated through efficient, timely and specific processes [3, 4]. The majority of these regulators belongs to the mitotic kinome, which encompasses kinase families and their counteracting phosphatases (or kinase inhibitors) [5]. Mass spectrometry analyses have identified more than 1000 distinct phosphoproteins, whose (de)phosphorylations are cell cycle-regulated. One-third of the mitotic proteins are phosphorylated on at least 10 distinct residues [6]. Phosphorylation activates mitotic proteins or maintains them in an inactive state. Mitotic enzymes are usually degraded through the E3 ubiquitin ligase APC/C (anaphase promoting complex/cyclosome) at the end of mitosis [7, 8].

Among other large kinase families, the mitotic kinome includes the Aurora kinases. Aurora members play key roles in mitotic entry, spindle assembly and cytokinesis. The “Aurora” denotation, reminiscent of the North Pole, comes from the monopolar spindles induced by *AURKA* gene mutations [9]. Aurora A (AurA) mostly controls centrosome maturation and bipolar spindle assembly, while Aurora B (AurB) and Aurora C (AurC) are required for condensation, attachment to kinetochores and alignment of chromosomes during (pro-)metaphase and cytokinesis [10–12].

## Evolution of the Aurora kinase family

**Fig. 1:**
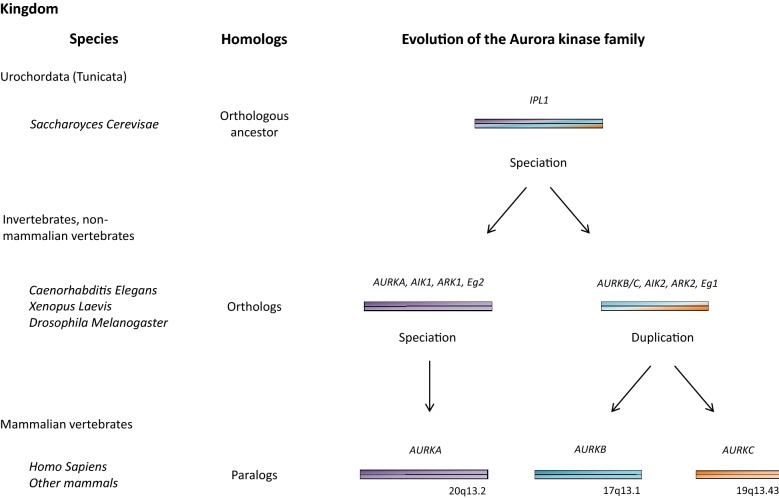
Evolution of the Aurora kinase family: Aurora members evolved from a single ancestor gene, called *Ipl1* and initially identified in *Saccharomyces cerevisiae* and other Urochordata (renamed Tunicata). In invertebrates and non-mammalian vertebrates (e.g. *Caenorhabditis elegans*, *Xenopus laevis* and *Drosophila melanogaster*), the Aurora family is constituted by two orthologs: the *AURKA* (also called AIK1, ARK1, Eg2) and the *AURKB* (also called AIK2, ARK2, Eg1) ancestor genes. In mammals, *AURKB/AURKC* ancestor gene duplication gave rise to *AURKB* and *AURKC* paralogs. In human, *AURKA*, *AURKB* and *AURKC* map on chromosomes 20q13.2, 17p13.1, and 19q13.43 respectively

The Aurora genes, relatively well conserved throughout evolution, are characterized by 78–84% of identity between human and rodent orthologs [13]. Phylogenetic trees suggest that Aurora members evolved from a single ancestor gene from *Urochordata* (i.e. *Tunicata*) (Fig. 1). The ancestral *AURK* gene, called *Ipl1* (Increased In Ploidy 1) has been identified in *Saccharomyces cerevisiae* and shares 41% of identity with the human *AURKA* gene. The orthologous evolution of *Ipl1* gave rise to *AURKA* and *AURKB/AURKC* ancestor genes in invertebrates and non-mammalian vertebrates, which were maintained during evolution.

In mammals, *AURKB* and *AURKC* genes are closely related paralogs, which evolved from the duplication of a common ancestral gene present in invertebrates and in non-mammal vertebrates (e.g. *Caenorhabditis elegans*, *Xenopus laevis* and *Drosophila melanogaster*) (Fig. 1) [9, 14–17]. Unlike other eukaryotes, mammals have a third *AURK* gene: *AURKC*, which plays the role of chromosome-passenger complex (CPC) protein in meiotically-active germ cells. *AURKB/AURKC* experienced the highest/lowest selection pressure during evolution [18]. In humans, *AURKA*, *AURKB and AURKC* map on chromosomes 20q13.2, 17p13.1, and 19q13.43 [3, 19].

## Structure of Aurora kinases

### Kinase domain

**Fig. 2:**
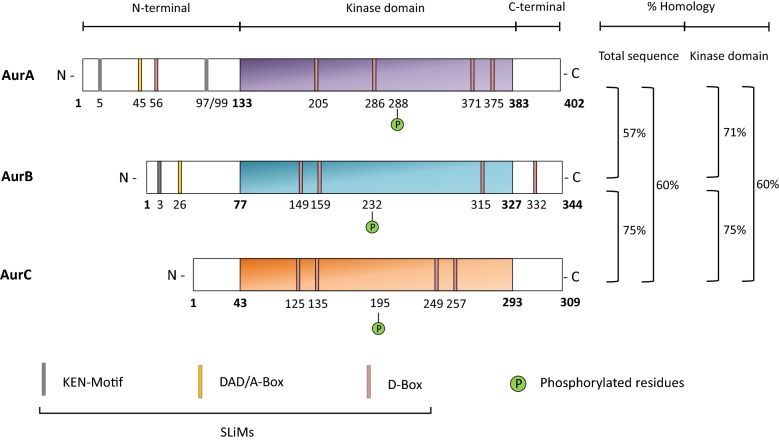
Structure of Aurora kinase domains. Aurora kinases are constituted by (i) an N-terminal domain, believed to control protein localization, (ii) a large and conserved catalytic domain containing the activation T-loop and (iii) a short C-tern domain with a D-box (Destruction Box). AurA and AurB also contain a KEN motif and an A-box that contributes to APC/C-dependent proteolysis. Numbers represent the residue position. Percentages of homology of the total amino acid sequence are evaluated (i) 57% between AurA and AurB, (ii) 75% between AurB and AurC and (iii) 60% between AurA and AurC. Percentages of homology of the catalytic domain are evaluated at (i) 71% between AurA and AurB, (ii) 75% between AurB and AurC and (iii) 60% between AurA and AurC

Aurora kinases contain a N-terminal domain (39–139aa), a kinase domain (250–300aa) and a C-terminal domain (15–20aa) (Fig. 2) [20]. The kinase domain is highly conserved between Aurora proteins, with 71%, 60% and 75% of homology between AurA/B, AurA/C and AurB/C, respectively. The Aurora kinase domain is constituted by a β-stranded N-terminal lobe and an α-helical C-terminal lobe that are linked together by a hinge region responsible for the active conformation [21]. The Aurora kinase domain is composed of twelve conserved subdomains, separated by less conserved insertion sites [22]. Table 1 describes the structural and functional properties of each of these catalytic subdomains (Table 1) [18, 21–23]. The C-terminal lobe of the kinase domain contains a conserved residue at Thr288 (AurA), Thr232 (AurB) and Thr195 (AurC), whose phosphorylation induces a conformation change associated to the acquisition of the kinase activity [24–27]. Aurora kinase domains carry SLiMs (Short, Linear Motifs), which are degrons for proteasome-mediated degradation (Fig. 2).

**Table 1:** Structure and role of the catalytic subdomains of Aurora kinases

Kinase domain lobe	Kinase subdomain	Structure	Role
N-terminal	I	Glycin-rich loop	ATP-binding
	II	Invariant lysine	
	III		
	IV		Structure of the N-terminal lobe
C-terminal	V		
	VIB		Mg^2+^ chelator
	VII	In front to the catalytic cleft	
	VIII		Substrate binding
	IX		Structure and active conformation of the kinase
	X		Substrate binding
	XI		Substrate binding
	XII		

The ATP-binding pocket of Aurora kinase domains, constituted by the binding of adenosine in a deep cleft between the two lobes, is also present in several other kinases, such as SRC kinase and GSK-3β [21]. However, the active site of Aurora kinases acquires a specific conformation after the folding of the N- and C-terminal domains. Notably, specific hydrogen bonds link the purine ring of adenosine to the hinge of the kinase domain of AurA [21]. Furthermore, sequence comparison of human Aurora proteins indicated the existence of three ATP-binding domain variants (L215, T217, R220), which were all specific to AurA [17]. The particularities of the AurA kinase domain turned out to be useful for the design of specific AurA inhibitors [21]. Moreover, the preferential activity of the antagonist, VX-680, to AurA (0.6 nM) over AurB (18 nM) and AurC (4.6 nM) supports the structural differences between AurA and AurB/AurC members [17].

### Non-catalytic domains

The N- and C-terminal domains of Aurora kinases can vary in size and sequence between human Aurora proteins and phylogenic homologs. The N- and the C- terminal domains contain degrons recognized by the Cdh1 protein (or Frz, in human) that trigger the degradation of Aurora proteins at the end of mitosis [28]. Cdh1 is a substrate-specific adapter that mediates E3 ligase APC/C activity after ubiquitination by the Ube2S E2 enzyme [18]. Three types of degrons exist in Aurora kinases: (i) the D-boxes, the first to be identified and the best characterized degrons, present in the kinase domain of all Aurora members, (ii) the KEN motifs present in AurA and AurB, whose role in proteasome-mediated AurA degradation is still controversial, and (iii) the DAD/A boxes, also present in AurA and AurB, identified as atypical degrons for Aurora degradation through APC/C (Fig. 2) [18, 29]. Evidence suggests that C-terminal D-boxes may play a role in intra-molecular interactions, rather than in APC/C-mediated degradation. In contrast, N-terminal D-boxes could make SLiMs degrons available prior APC/C-mediated degradation [29].

In parallel, the C-terminal domain of Aurora kinases mediates co-factor interactions responsible for protein conformation and, thus, Aurora kinase activity. The N-terminal domain, highly variable between Aurora members, may direct Aurora kinase binding to protein partners that direct their cellular localization [12, 30]. Interestingly, AurA and AurB mitotic functions can rescue each other according to their subcellular localization [31]. AurC, whose expression is restricted to germ cells, can also rescue the role of AurB as CPC enzyme when in somatic cells [32]. A growing body of evidence suggests that the specialized functions of Aurora kinases in mammalian cells are mainly mediated by their subcellular or cellular localization.

## Regulation of Aurora kinases

### Transcription

**Fig. 3:**
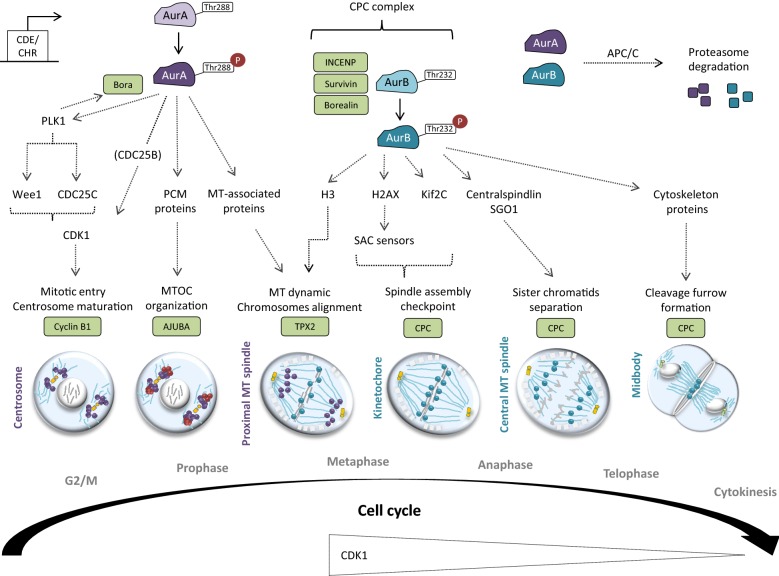
Role of AurA and AurB in mitosis. The cell-cycle dependent transcription of AurA and AurB are under the control of the CDE/CHR elements, which are recognized by the E4TF1 transcription factor. AurA is mainly activated after Thr288 auto-phosphorylation. Bora, a key AurA co-factor, is phosphorylated by AurA and, in return, Bora enhances the kinase activity of AurA. Once activated, AurA phosphorylates and activates CDK1-Cyclin B to allow G2/M checkpoint unlock through various mechanisms, including: (i) PLK1-dependent targeting of Wee1 and CDC25C, (ii) CDC25B-dependent activation of CDK1 and (ii) direct phosphorylation of CDK1. Then, PLK1 mediates Bora degradation to permit mitosis progression. At G2/M, AurA localizes in the centrosome and also contributes to their maturation before mitotic entry. At prophase, AurA—whose activity is maintained by Ajuba- recruits and phosphorylates several PCM proteins (i.e. γ-TuRC, centrosomin, NDEL1, TACC, LATS2 and BRCA1) to organize the MTOC. At metaphase, AurA moves to the proximal MT and targets MT-associated proteins (i.e. Ki2a, TACC3, CKAP5-a) to organize the mitotic spindle. At this time, TPX2 allows the maintenance of the activate state of AurA. AurB binds INCENP, Survivin and Borealin to form the CPC complex and to be activated upon Thr232 auto-phosphorylation. AurB, firstly localized on chromosomes, contributes to their proper alignment at metaphase. Prior anaphase, AurB concentrates to the kinetochore to allow the spindle assembly checkpoint (SAC) crossing through (i) H2AX-dependent activation of SAC sensors and (ii) Kif2C recruitment. Then, AurB moves to the central MT to trigger sister chromatids separation through Centralspindlin and SGO1 recruitment at anaphase. Finally, AurB targets various cytoskeleton regulatory proteins (RhoA, Vimentin, Desmin, GFAP) at the midbody in order to organize the cleavage furrow for cytokinesis. At the end of mitosis, both AurA and AurB undergo ubiquitination and proteasome degradation by APC/C, which happen subsequently to their dephosphorylation by PP2A or PP1

The cell cycle-induced transcription of Aurora proteins is made possible by the presence of CDE (cell cycle-dependent element) and CHR (cell cycle gene homology region) in *AURK* promoters. These CDE/CHR sequences in *AURKA* mediate the transcription of AurA and other crucial G2/M regulators (e.g. Cyclin A, CDC25C, CDK1 and PLK) (Fig. 3) [33–35]. For AurB, transcription is induced upon binding of E2F-1, E2F-4, DP-2 and FoxM1 transcription factors with the CDE/CHR sequences within the *AURKB* promoter during prophase [36].

Like other Aurora members, AurC expression is cell-cycle regulated and is increases during the meiotic phases of germ cells. In the testis, AurC transcription is regulated by a PLZF (Promyelocytic leukaemia zinc finger protein)-related transcription factor, Tzfp (testis zinc finger protein) [37], which is responsible for specific AurC expression in germ cells. In mouse testes, *AURKC* mRNA appears 14 days after birth and starts to decrease from day 28 [34, 37–39]. In oocytes, *AURKC* mRNA is recruited by CPE (cytoplasmic poly-adenylation element), the “maternal signal” which ensures meiosis completion during early development [40]. In somatic cells (i.e. muscle, placenta, lung and bladder), AurC expression is hardly detectable, restricting it to meiotically-active germ cells. Although AurC transcription is similar to AurB, whether AurB transcription factors control AurC expression still needs to be determined [37, 41].

### Post-translational modifications

Each Aurora member becomes phosphorylated at specific residues upon co-factor binding during mitosis. Like most of eukaryotic kinases, Aurora proteins acquire an active kinase conformation thanks to the activation loop [42]. This active kinase conformation is acquired upon auto-phosphorylation through an intermolecular (*trans*)-reaction within the two-lobed Aurora kinase domain. In such a reaction, the two catalytic lobes form an asymmetric dimer in which one monomer acts as the active enzyme and the other as the substrate [43]. Aurora kinase activity is induced by auto-phosphorylation of conserved threonine residues (i.e. the Thr288 (AurA), Thr232 (AurB) and Thr195 (AurC) residues), co-factor bindings and recruitment at mitotic structures (Fig. 3).

AurA auto-phosphorylation is mediated by several co-factors acting at different steps of mitosis, the best known being Ajuba, TPX2 (Targeting protein for Xenopus kinesin-like protein 2), Bora (protein aurora borealis) and TACC3 (Transforming acidic coiled–coil-containing protein 3). The initial AurA^Thr288^ phosphorylation causes a positive feedback phosphorylation loop responsible (i) for maintenance of the activated state of AurA until anaphase and (ii) for the AurA activity peak from late G2 until pro-metaphase (Fig. 3).

During prophase, AurB phosphorylates INCENP (Inner Centromere Protein), the scaffolding component of the CPC. In return, binding to INCENP triggers AurB auto-phosphorylation and changes its conformation to induce kinase activity [11, 44]. AurB then binds additional protein subunits, mostly the Survivin and the Borealin proteins, to form the CPC. The integration in the CPC is required for AurB kinase activity and localization throughout cell division [45–47]. AurC phosphorylates the same substrates as AurB (i.e. INCENP, Survivin, Borealin) and is submitted to similar regulation as AurB in meiotically-active cells (Fig. 3) [48].

Interestingly, AurA can also be phosphorylated by upstream kinases at Thr288 residue (e.g. mTOR, PAK-1, PKA) and on other residues that activate (Thr287, Ser283/284) or inhibit (Ser342) its kinase activity [49, 50]. Thr287 phosphorylation seems to be an alternative or compensatory regulation to Thr288 phosphorylation in AurA activation. AurA can also be regulated by Ca^2+^/Calmodulin (CaM), which induces its auto-phosphorylation at Ser51, Ser53/54, Ser66/67 and Ser98 residues. Similarly, the nucleolar protein Nucleophosmin (NPM) acts as chaperone to activate AurA via Ser89 phosphorylation in the centrosome. NPM-mediated phosphorylation of AurA occurs only if AurA is previously phosphorylated at Thr288 residue [51]. These specific phosphorylations may be involved in protecting AurA against degradation until the end of mitosis [52, 53]. In addition, AurA acetylation at K75/K125 by ARD1 (Arrest Defective Protein 1) acetyl-transferase favors its kinase activity [54]. Nevertheless, Thr288 auto-phosphorylation seems to prevail over any other regulation modes of AurA or to be the pre-requisite for full AurA activation [43]. AurA becomes inactive if dephosphorylated at Thr288 residue. However, the Thr288 phosphorylation does not always reflect an active state of AurA. This suggests thus that the Thr288 phosphorylation is a necessary but insufficient condition for full AurA activation, which could require additional post-translational modifications. The presence of multiple phosphorylation sites in AurA, as well as several upstream kinases able to target AurA, reflect a longer speciation of AurA throughout evolution, as suggested by phylogenetic analyses.

### Degradation

Aurora degradation is mediated by APC/C and activated by the Cdh1 protein, which recognizes degrons present in Aurora proteins. Once activated, APC/C ubiquitinates Aurora and initiates its proteasome-mediated degradation [55, 56]. AurA degradation starts during the mitotic phase and is completed during G1, while AurB and AurC proteins are degraded after cytokinesis [57]. Aurora protein ubiquitination can be induced by dephosphorylation at Ser51 residue by the PP1 or the PP2A phosphatases [58]. Furthermore, the APC/C-mediated degradation of AurA is more efficient than AurB, with AurC being the most stable Aurora member [29, 59]. AurB also binds the MT-binding protein EB1, which protects AurB against degradation [60].

The distinct expression pattern of AurB and AurC may result from post-transcriptional regulation of *AURKC* mRNA in germ cells, which enhances protein stability during germ cell maturation [40]. Indeed, germ cells contain both AURKB and AURKC mRNA, but the amount of AurB protein declines during meiosis and early embryonic mitosis, while the amount of AurC is maintained during pre-implantation development [40]. Interestingly, AurC overexpression in the centromeres of tumor cells leads to a decreased level of AurB, suggesting a negative feedback loop between the two kinases [41].

## Cellular and subcellular localization

AurA and AurB are constitutively expressed in mitotically active cells and up-regulated in highly proliferative tissues. AurC expression is restricted to germ cells of both genders during meiosis. The specific subcellular localization of each member varies throughout mitosis progression.

Low levels of AurA proteins are diffusely distributed throughout the cytoplasm of interphase cells and often enriched in the MT [61]. During the G2 phase, AurA transcription increases and accumulates within cell nuclei due to a Nuclear Localization Sequence (NLS), localized in the N-terminal domain [62]. After nuclear envelope breakdown (NEBD), AurA is released throughout the cytoplasm. Then, AurA mainly concentrates in centrosomes and in the proximal mitotic spindle. AurA targeting to the centrosomes is enabled by the N-terminal domain in a microtubule (MT)-dependent manner in both mitotically-active and inactive cells [63]. Depletion of the N-terminal domain alters spindle formation, chromosome alignment and mitotic onset [31, 63]. While the catalytic C-terminal domain is able to target AurA to the centrosome, It is to a lesser extent and is independent of the MT array [64]. Moreover, the C-terminal domain is crucial for spindle bipolarity during mitotic progression, but not for mitotic onset. These suggest that the predominant role of the N-terminal domain in AurA localization is on the onset of mitosis; whereas, the C-terminal domain impacts mitosis progression by altering AurA conformation and kinase activity.

AurB is recruited in the nucleus of interphase cells thanks to a NLS sequence localized in its N-terminal domain. In contrast, AurB localization during mitosis is regulated by its C-terminal domain [65]. During prophase, Survivin targets AurB to the centromeres to mediate the function of the CPC in proper kinetochore attachment. In (pro-)metaphase, AurB localizes at chromosomes of the midzone to trigger spindle elongation and stabilize cleavage furrow [66]. AurB delocalization to the midzone is regulated by direct interaction between MT, Borealin and the putative coiled–coil (CC) domain of INCENP.

Interestingly, the kinase activity of AurB during mitosis—but not cytokinesis—can be rescued by the catalytic domain of AurA [67]. The depletion of AurA does not affect the levels of AurB, which may suggest the absence of any compensatory process [63]. However, the targeting of AurB at the centrosomes mediates the function of AurA in mitotic entry. Inversely, AurA expression at chromosomes rescues the role of AurB as a CPC protein [3, 31]. These suggest that the specific localization of AurA and AurB may dictate the binding to distinct partners or substrates that specify their function.

The sparse data about AurC localization during meiosis report a similar distribution pattern to AurB in mitotic cells [48]. AurB and AurC are also similar in terms of sequences, substrates and functions during cell division. The only apparent difference concern AurC localization in interphase germ cells, which involves centrosomes rather than nuclei. However, forced expression of AurC in somatic cells can rescue the role of AurB in the phosphorylation of Histone H3 and in chromosome segregation [11, 67, 68].

To summarize, the mitotic roles of each Aurora member seem to mostly rely on their expression, temporal restriction and localization, rather than on their kinase activity [31]. On the other hand, non-mitotic roles of Aurora kinases are not yet shown to depend on their localization in interphase cells.

## Somatic cells

### Centrosome maturation

Before spindle assembly, AurA favors centrosome maturation that involves MT nucleation and peri-centriolar material (PCM) recruitment to the MT-organizing center (MTOC). The increase of CDK11 (cyclin-dependent kinase 11) in prophase contributes to AurA and PLK1 (polo-like kinase 1) recruitment at centrosomes [69]. The targeting of AurA at centrosomes is also mediated by Src, which is responsible for Golgi apparatus cleavage before G2 [70]. Once localized at centrosomes, AurA binds and phosphorylates the LIM-domain-containing protein Ajuba that, in turn, favors the Thr288 auto-phosphorylation and centrosome maturation (Fig. 3). Ajuba binds the N-terminal domain of AurA, which directs its targeting at centrosomes [63]. Additional scaffolding proteins (such as CEP192, NEDD9, PAK and NPM) and other binding partners (Arpc1b, LIMK1, PP1 inhibitor) also favor AurA phosphorylation and centrosome maturation [50, 51, 71, 72].

The role of AurA prevails in centrosome growing, rather than in their duplication [73]. Once fully activated, AurA recruits γ-TuRC (γ-tubulin ring complex) and Centrosomin, which are required for the elongation and nucleation of MT (Fig. 3). AurA also phosphorylates and recruits TACC, NDEL1 (Nuclear distribution protein nudE-like 1), LATS2 (large tumor suppressor 2) and BRCA1 (breast cancer type 1) to the MTOC. The phosphorylated form of TACC binds the MT-binding protein CKAP4 (Cytoskeleton-associated protein 4) to stabilize the minus end of the MT at centrosomes and to organize the actin cytoskeleton. LATS2 and BRCA1 mediate γ-tubulin recruitment and, thus, MT nucleation during centrosome maturation [10, 74–79].

Simultaneously, AurA activates and targets the Cyclin B1-CDK1 complex at centrosomes [80]. AurA can stimulate the Cyclin B1-CDK1 complex either by direct phosphorylation or by phosphorylation of the Cyclin B1-recruiting CDC25B (M-phase inducer phosphatase 1) phosphatase. On the other hand, AurA phosphorylates PLK1, which represses the Wee1 inhibitor and activates CDC25C responsible for CDK1 activation. This first wave of Cyclin B1-CDK1 activation is required for the transition from the G2 to the M phase (Fig. 3).

In late G2, AurA phosphorylates Bora, itself targeted in the cytoplasm in response to CDK1 up-regulation [81]. Moreover, the binding of AurA to Bora induces an increase of AurA kinase activity. AurA phosphorylates PLK1 which is crucial for mitotic entry, centrosome cycle, spindle assembly and cytokinesis (Fig. 3) [80, 82]. PLK1 also phosphorylates Bora, thereby recognized by the SCF^β−TrCP^ (Skp, Cullin, F-box containing complex, β-transducin repeat-containing protein) ubiquitin ligase and degraded by the proteasome [83]. Bora degradation makes AurA available for coupling with TPX2, a MT-associated protein (MAP) essential for the maintenance of AurA activity.

### Establishment of the bipolar spindle

After centrosome maturation, centrosomes are segregated at the cell poles to establish the bipolar spindle. Monopolar spindles are systematically observed in AurA-deficient cells, suggesting a possible role of AurA in centrosome separation [76, 80, 84]. Indeed, AurA contributes to centrosome separation via phosphorylation of kinesin Eg5, which regulates the anti-parallel forces of the spindle MT. Moreover, AurA balances the cycles of MT assembly and disassembly to control mitotic spindle dynamics. Notably, AurA (i) inhibits the Kif2a (Kinesin Family Member 2A) MT depolymerase, (ii) recruits TACC3, which in turn, induces MT growth through CKAP5-a (Cytoskeleton-associated protein 5-A) and (iii) stabilizes MT around the centrosomes by antagonizing Kif2C (Kinesin Family Member 2C) (Fig. 3) [79, 84–89].

A second wave of Cyclin B1-CDK1 phosphorylation by AurA occurs in late prophase. At this time, Cyclin B1-CDK1 activation induces the release of spindle assembly factors (i.e. TPX2) and activates the Ran GTPase pathways responsible for NEBD. TPX2, localized at the spindle poles, binds the AurA catalytic domain and confers it an active conformation. This active conformation allows (i) AurA localization on the astral MT, (ii) Thr288 auto-phosphorylation and (iii) prevention of the dephosphorylation of AurA by the PP1 and PP6 phosphatases. The TPX2-AurA complex thereby constructs the bipolar mitotic spindle during cell division [7, 11, 80, 90–92].

### Chromosome alignment and kinetochores anchoring

At the beginning of mitosis, AurB phosphorylates the Ser10 residue of histone H3, leading to the release of heterochromatin protein 1 (HP-1) from heterochromatin and to an epigenetic switch toward an active chromatin conformation [93]. The H3 phosphorylation may favor chromosome condensation and/or AurB recruitment to the centromeres (Fig. 3) [35, 46, 94–96]. Although its functional impact is not completely understood, H3 phosphorylation at Ser10 residue is now commonly used in AurB kinase assays. Interestingly, AurC can also trigger histone H3 phosphorylation in mitotic and meiotic cells, suggesting an overlap between AurB and AurC [48].

During prophase, AurB concentrates in the kinetochores to mediate attachment between chromosomes and MT. AurB-mediated phosphorylation of the H2AX histone in the kinetochores promotes AurB auto-phosphorylation and activation [97]. AurB then allows chromosome bi-orientation through the regulation of the spindle assembly checkpoint (SAC). The SAC, activated by checkpoint sensors (BUB, CENP-E, MAD-1/2 and MPS1 proteins), detects unattached or poorly attached kinetochores. Notably, AurB recruits and phosphorylates Kif2C to depolymerize the incorrectly attached kinetochores. Furthermore, AurC phosphorylates the Centromere Protein A, required for the recruitment of additional kinetochore proteins prior chromosome segregation. The passage of the spindle assembly checkpoint is a signal for APC/C activation, which marks the completion of mitosis (Fig. 3) [8, 98].

### Chromosome separation

AurB and other CPC component are recruited to the centromeres and to the midzone to regulate chromosome separation. The targeting of the CPC at the centromeres is triggered by the CULIN3-containing ubiquitin ligase and is negatively regulated by the Cyclin B1-Cdk1 complex. Cyclin B1 levels start to decrease from metaphase, leading to a rapid drop of Cdk1 activity (Fig. 3). During (pro-) metaphase, AurB and other CPC components are recruited to the midzone, partially by the MKLP2 kinesin (Mitotic Kinesin-Like Protein) regulating MT dynamic [11, 99–103]. The formation of the central spindle prior anaphase relies on the Kinesin-like protein KIF23 and the Rho GTPase-activating protein RacGAP1 (Rac GTPase-activating protein 1).

AurA was recently shown to be required for central spindle assembly during later mitotic steps, i.e. during anaphase [104, 105]. Indeed, AurA depletion triggers MKLP1 delocalization and accumulation of the MAP DCTN1 (dynactin subunit 1) at spindle poles, which impairs bidirectional transport along MT and central spindle assembly [104].

From metaphase to anaphase, AurB has been shown to control centromeric and telomeric heterochromatin segregation in fission yeast [99, 106]. Indeed, telomeric AurB allows telomere dispersion through dissociation of HP-1 and Rad21, the cleavable component of the Cohesin complex. AurB phosphorylates Cnd2 (Condensin Complex subunit 2), which enhances chromosomes condensation and full telomere disjunction [106]. Furthermore, AurB recruits SGO1 (Shugoshin 1) to the centromeres to remove Cohesin and to trigger sister chromatid separation during anaphase.

### Telophase and cytokinesis

The regulation of HP-1 by AurB is crucial for the maintenance of genome integrity during telophase. Furthermore, AurB locally prevents nuclear envelope assembly to facilitate the incorporation of late-segregating acentric chromosomes, likely to form damage-prone micronuclei [107].

During cytokinesis, AurB localization is maintained to the midbody to activate the RhoA GTPase after RacGAP1 phosphorylation [8, 47]. AurB thereby induces actin polymerization and myosin activation, both required for the formation of the contractile ring during cytokinesis. Moreover, AurB phosphorylates additional substrates (i.e. Vimentin, Desmin and GFAP) to organize the cleavage furrow (Fig. 3) [11, 44]. AurB thus plays key roles in the maintenance of genome integrity during telophase and in the segregation of cytoplasmic components during cytokinesis.

### Mitotic checkpoint

Mitosis of non-cancerous cells whose DNA is damaged (i.e. by UV, γ-irradiation or chemicals) can be paused or cancelled at the cell cycle checkpoints (G1/S/G2). These checkpoints regulate the fates of cells with altered DNA, which are (i) DNA repair, only possible during the cell cycle pauses at the checkpoints, (ii) entry into the senescent state (G0) of G1-frozen cells, whose DNA cannot be repaired, or (iii) apoptosis. The G1 checkpoint prevents the replication of altered DNA and, thus, the transmission of mutations to daughter cells. The S checkpoint stops mitotic progression before the end of DNA synthesis, while the G2 checkpoint prevents mitotic entry in the case of double strand breaks (DSB) in the replicated DNA. DSBs are detected by sensor proteins (i.e. Rad50, Mre11, Nbs1), which themselves stimulate ATM (ataxia telangiectasia mutated) and ATR (ATM-and Rad3 related) kinases. DSB can be repaired by (i) homologous recombination (HR), a highly accurate process using sister chromatids as templates for DNA replication, or by (ii) non-homologous end joining (NHEJ) mediated by excision repair enzymes more likely to make errors ATM and ATR phosphorylate and activate Chk1/Chk2 (checkpoint protein 1/2), which sequestrate Cdc25 in the 14-3-3 scaffold protein. Cdc25 sequestration prevents Cyclin B1 recruitment, leading to G2/M blockade. On the other hand, ATM/ATR also induce the p53-dependent transcription of p21 and Gadd45 (Growth arrest and DNA damage-inducible protein GADD45 alpha), which inhibit CDK1-induced mitotic entry [108].

In response to DNA damage, Chk1 represses AurA to block the cell cycle before mitotic entry. If DNA can be repaired, the checkpoint must be overridden to proceed into mitosis. Mechanisms of checkpoint recovery involve the inhibition of Chk1, allowing the reactivation of AurA. During checkpoint recovery, AurA reactivation is followed by stimulation of PLK1 and CDC25B [82, 92, 109]. Interestingly, centrosomal p53 proteins inhibit AurA via transcriptional (via regulation of E2F3 by p21) and post-transcriptional (via ubiquitination and Ser215 phosphorylation) regulation [110]. In cancer, AurA can thereby mediate chemo- and radio-resistance through efficient HR-dependent DSB repair [111].

On the other hand, AurB is also down-regulated in response to irradiation through ATM-dependent dissociation of PP1 (protein phosphatase 1) [112]. AurB inhibition is associated with Chk1 activation in mitotic cells with a delayed H3 phosphorylation, reflecting a delayed chromosome replication and condensation [113]. AurB is also involved in the NHEJ pathway to repair DNA damage. Notably, the Ku heterodimer (Ku70/Ku80), a scaffolding protein of the NHEJ complex, represses AurB kinase activity after irradiation [114]. In response to DNA damage, PARP1 (poly(ADP-ribose) polymerase 1), a chromatin-associated DNA repair enzyme, also antagonizes AurB to block mitosis and histone H3 phosphorylation [115].

## Stem cells

### Asymmetric cell division, polarity and migration

Asymmetric cell division (ACD) allows the asymmetric segregation of mother constituents that specify the fate of daughter cells toward a self-renewing copy of the mother cell or a differentiated cell [116]. In *Drosophila* neuroblasts (NB), Bora is recruited by the Cyclin B1-CDK1 complex and activates AurA required for asymmetric polarization [7, 81]. AurA monitors cell polarization through phosphorylation of PAR6 (Partitioning defective protein 6), responsible for aPKCζ (atypical Protein Kinase Cζ) activation and Numb segregation. Numb is a cell fate determinant that induces cell differentiation through Notch inhibition [117]. Then, AurA regulates the Pins/LGN/Dlg complex that allow the asymmetrical establishment of the bipolar spindle [117–121].

In embryonic stem cells (ESC), AurA loss affects self-renewal and triggers differentiation by p53 activation [12, 122]. AurA inhibition alters ACD and, thus, the stemness potential of ESC. However, AurA does not alter ESC proliferation and viability, both mediated by symmetric divisions. In contrast to somatic cells, stem cells do not require AurA-mediated centrosome maturation to divide. AurA thus triggers independent molecular mechanisms in somatic cells and stem cells to achieve proper cell division.

On the other hand, AurB is required for the maintenance of telomeres in stem cells through the phosphorylation of histone H3, which is able to remodel chromatin at telomeres. Indeed, in ESCs, AurB controls the structural integrity of telomeres and maintains telomeric repeats at chromosome ends through regulation of the reverse transcriptase telomerase [93, 123].

Increasing evidence suggests that AurA plays non-mitotic roles in cell polarity through MT dynamics via the NDEL1 MAP, crucial for MT organization, intracellular trafficking and cell motility. In migrating neurons, aPKC activates AurA which, in turn, recruits NDEL1 and promotes neurite elongation by modulation of MT [124]. Similarly, AurA also regulates MT organization required for neuronal migration during cortical development [125].

### Embryonic development

AurA deregulation has major functional impact during embryonic development. AurA inhibition in mouse embryonic fibroblasts (MEFs) leads to aneuploidy due to defects in mitotic onset and spindle assembly. AurA knock-out (AurA^−/−^) mouse embryos die during early developmental steps, i.e. at 10.5 days post coitum (d.p.c) [126]. In the same study, AurA^+/−^ mice develop more tumors compared to wild-type (WT) mice. Another study showed that AurA^−/−^ mouse embryos exhibited reduced cell growth and division, disorganized spindle, misaligned chromosomes and micro-nucleated cells at 3.5 d.p.c and lethality before 8.5 d.p.c [127]. The hypothesis is that AurA-null embryonic cells undergo a minimum of four mitotic divisions, made possible through residual maternal AurA protein in oocyte and zygote [128]. Another hypothesis is that AurA depletion may delay mitosis but does not completely prevent mitotic entry. Indeed, the proportion of abnormal mitotic cells increases during the development of AurA^−/−^ mouse embryos [127]. Furthermore, AurA depletion in *Xenopus laevis* egg extracts retards Cyclin activation, DNA condensation, and bipolar spindle formation during mitosis progression but allows mitotic entry [63].

Although AurB-deficient implanted embryos exhibit pro-metaphase figures and apoptosis, the role of AurB as a CPC is dispensable for early embryonic divisions [68]. The hypothesis is that AurC may also rescue the role of AurB during embryogenesis. Indeed, AurC expression is specific in pre-implantation embryos and declines in late blastocyst stages [68]. Like AurB, AurC accelerates blastomere division in 2-cell embryos and induces the expression of pluripotency-related genes [129]. During mitotic embryonic divisions, AurB then relays the predominant function of CPC and maintains this role in mature somatic cells [48].

### Germ cells

Meiosis is composed of two steps: (i) Meiosis I (MI), which produces two haploid cells (n = 1) from one diploid cell (n = 2), and during which homologous chromosomes separate and, (ii) Meiosis II (MII), during which sister chromatids separate to give rise to four haploid cells. During MI, AurA auto-phosphorylation induces *Mos* mRNA adenylation and transcription by CPEB (Cytoplasmic Polyadenylation Element Binding protein). Mos is a germ cell-specific serine/threonine kinase, responsible for MAPK activation and entry in meiosis. AurA thereby induces entry into meiosis I, as well as proper spindle formation and germinal vesicle breakdown (GVBD) [130, 131]. AurA phosphorylation then drops during interkinesis and rises again during entry in MII. Interestingly, oocytes injected with AurA blocking antibodies, which did not interfere with kinase activity, fail to organize the metaphase plate and to undergo MII. AurA may thus play a role in MII onset independently of its kinase activity [130, 132].

AurB protein levels rapidly decrease during meiosis and its CPC function can be partially compensated by AurC. However, AurB and AurC are reported to play different roles during meiosis. *AURKB*-deficient mice die at the blastocyst embryonic stage, and while *AURKC* knock-out mice are viable, they are infertile due to cytokinesis defects [48]. Moreover, *AURKB* mutation in mice heavily affects spermatogenesis and sterility, suggesting that AurC is unable to fully compensate for AurB alteration [32, 40]. On the other hand, oocytes overexpressing *AURKB* fail to activate APC/C and exhibit chromosome segregation defects. Meanwhile, *AURKC* up-regulation in germ cells causes MI arrest due to similar chromosome segregation defects and cytokinesis failure [34, 37–39, 133]. Interestingly, *AURKC* mutation is a leading cause of macrozoospermia, in which sperm cells exhibit large blunted heads which contain extra acrosomes [32].

## Tumor cells

### Expression and clinical significance

The mechanisms of AurA-mediated tumorigenesis is supported by a large body of work, in contrast to AurB/C, whose oncogenic activities are not yet fully understood [48, 134–136]. Amplification, overexpression or hyper-activation of AurA is found in several aneuploid tumors including breast, colorectal, gastric and prostate cancers [134, 137, 138]. Moreover, AurA expression predicts patient prognosis in various cancers including breast, colorectal, nasopharyngeal and gastric tumors [139–142].

AurB is also found up-regulated in most aneuploid human tumors, but it is debatable if AurB overexpression transforms non-cancerous cells or results from tumor cell over-proliferation. Nevertheless, AurB overexpression is an independent poor prognosis factor which correlated with worse histo-pathological characteristics (i.e. tumor stemness, invasion, proliferation) in hepatocellular carcinoma, non-small cell lung carcinoma and, oral squamous cell carcinoma [143–145].

AurC is a cancer-testis antigen (CTA), which is expressed in meiotically-active germ cells and aberrantly found in some cancer cells lines and in human thyroid carcimomas, cervical and colorectal tumors [36]. In colorectal cancer, AurC protein level is positively correlated with the stage of malignancy, Although AurC overexpression was shown to transform somatic cells and favor tumor progression, the mechanisms of AurC-mediated tumorigenesis are not yet described [41].

### Aneuploidy

AurA overexpression can lead to centrosome overgrowth, multipolar spindle formation, unequal chromosome segregation, and aneuploidy in mitotically active cells, which can thereby degenerate into pre-cancerous cells. The role of AurA in aneuploidy is mostly mediated by p53, BRCA1/2 and RASSF1A (Ras association domain-containing protein 1) [73, 78, 111]. On the other hand, the inhibition of AurA also causes aneuploidy due to monopolar mitotic spindles, chromosomal separation defects and chromatin bridges [146]. In stem cells, AurA loss affects ACD and self-renewal rather than mitosis, proliferation or survival. Thus, AurA is believed to play a marked tumor suppressive activity in stem cells [12]. Indeed, AurA loss disturbs ACD of *Drosophila* NB, leading to forced symmetric division and over-proliferation [117]. The implantation of a larval brain fragment of AurA-dead mutants in normal flies leads to abdominal tumors that can be fatal [147]. Conversely, AurA overexpression in *Drosophila* NB also promotes tumorigenesis due to centrosome dysfunction and aneuploidy [119]. Thus, unbalanced AurA levels are likely to transform stem cells through alteration of their mode of division.

Similar to AurA, AurB overexpression enhances aneuploidy, genetic instability, and risk of malignant transformation. AurB-overexpressing cells remain in mitosis for longer periods, probably through inhibition of p21Cip1, a cyclin-dependent kinase inhibitor. AurB alteration impairs chromosome segregation, SAC activation, and, thus, proper cell division [136]. As already mentioned, the role of AurB in maintaining genome integrity could also result from HP-1 regulation and micronuclei formation during telophase [107].

AurC also triggers centrosome overgrowth and multi-nucleation, induces cell transformation and, thus, favors tumor initiation [41, 148]. However, the significance of AurC activity in oncogenic transformation should be supported by additional studies.

### Proliferation and survival

In tumor cells, AurA interacts with alternative oncogenic pathways, such as Myc, PKC/MAPK, BCR/ABL, NFκβ or Wnt/β-catenin pathways, to favor cell proliferation, survival and therapeutic resistance [27, 149]. AurA also modulates pro-apoptotic (BCL2, MLC1) and anti-apoptotic (Bax, Bim, Puma, Apaf) proteins to confer a survival advantage to tumor cells [27]. Moreover, AurA is a central regulator of the Pi3 K/Akt pathway, which favors malignant transformation and resistance to anti-cancer therapies [150].

On the other hand, AurB activates CDK1 in a p53-dependent manner and inhibits Caspase-3 expression, leading to tumor cell proliferation and survival [151, 152]. In contrast, only few studies report the role of AurC in tumor progression [48]. Khan et al. showed that AurC overexpression in Hela cells improved tumor initiation and growth in subcutaneous mice xenograft experiments [41].

### Stemness, epithelial-to-mesenchymal transition and migration

Several studies indicated that AurA is a key modulator of cancer stem cells (CSCs) in gliomas, breast and colorectal tumors [153, 154]. CSCs represent a small cell population of the tumor, which possesses stem cell-like properties (i.e. self-renewal, multipotency) and marked tumor-initiating ability [27, 155–157]. In breast cancer, AurA reprograms Smad5-dependent transcription to enhance tumor stemness, epithelial-to-mesenchymal transition (EMT), and breast tumor metastases [27, 155–157].

EMT is mediated by the loss of adhesion molecules, which confers a migratory advantage to tumor cells. In various tumors, AurA triggers EMT via direct transcriptional activation of Twist, Slug and, Zeb or via indirect activation of the Wnt/Akt pathway, which controls methylation of the Twist promoter [157–159]. AurA also enhances tumor cell migration through several pathways. For example, AurA activates the Cofilin-F-Actin pathway responsible for breast cancer metastases [160]. In esophageal carcinomas, AurA triggers tumor cell migration through MMP-2 (matrix metalloproteinase 2) secretion via p38/Akt pathway activation. AurA can also stimulate the DNA-binding protein Rap1, a member of the Ras GTPase family, to favor oral carcinoma metastasis [160–162]. In head and neck squamous cell carcinoma (HNSCC), AurA favors cell migration and invasion through FAK (Focal Adhesion Kinase) activation via the Akt pathway [163]. Moreover, chemical inhibition of AurA, but not AurB, impairs with MT dynamics (i.e. shrinkage, growth rate, frequency rescue and nucleation) during interphase of Hela cells [164].

Interestingly, high nuclear *AURKA* expression favors breast cancer stemness and is associated with poor patient prognosis [165]. Furthermore, AurA is delocalized from the nucleus to the cytoplasm in mitotically-inactive cells, as well as in migrating cells [166]. Altogether, those data suggest that disrupted AurA localization, observed in most AurA-overexpressing tumors, may mediate oncogenic activities of AurA [167].

## Conclusion and perspectives

During mitosis, AurA and AurB allow mitotic spindle assembly through MT nucleation at centrosome and MT stabilization around chromosomes. AurA loss induces monopolar spindles and aneuploidy, while AurB deficiency alters chromosome distribution and cytokinesis resulting in bi-nucleated cells [9, 146]. AurA and AurB share several targets crucial for spindle bipolarity and their loss disrupt chromosome alignment. Both kinases control common mitotic processes from the opposite ends of the spindle MT [3, 61, 168]. The role of CPC is mostly mediated by AurB in somatic cells and by AurC in germ cells, although each member plays some non-overlapping functions during embryonic development. In view of these data, Aurora kinases may benefit from two levels of regulation: (i) the kinase activity induced by auto-phosphorylation and interaction with specific partners, and (ii) the subcellular localization, which is regulated by the N- and C-terminal domain of Aurora proteins. Interestingly, Aurora kinase activity of Aurora proteins depends on sub-cellular localization, indicating the importance of Aurora protein localization at the functional level.

Likely resulting from their role in mitosis, AurA and AurB are overexpressed in most of aneuploid tumors, which account for 90% of human malignancies [2]. AurA and AurB turned out to be promising prognosis factors for cancer patients, while the oncogenic roles and prognosis value of AurC should be further investigated. In contrast to AurB, AurA can trigger neoplastic transformation and play more diversified oncogenic functions, some of them being unrelated to mitosis [136, 152].

Interestingly, the *AURKA* gene is the member of the Aurora family which has undergone the longest speciation throughout evolution [18]. Compared to other Aurora members, AurA exhibits multiple kinase domain variants, numerous degrons and phosphorylable residues which can also be targeted by exogenous kinases [18, 21]. It is conceivable that the newly-acquired oncogenic functions of AurA may result from a stronger specification during evolution.

Recent data indicated that the oncogenic activities of Aurora proteins may rather depend on their (sub-)cellular localization, which dictates their role during mitosis of non-cancerous cells [31]. Indeed, Aurora proteins share a highly conserved kinase domain and more distinct non-catalytic N-terminal and C-terminal domains [21]. During mitotic progression, AurB and AurC are mostly involved in chromosome function, which may explain their role in aneuploidy and genetic instability. The localization of AurA in MT may explain why AurA up-regulation is responsible for both survival and motility of tumor cells [169]. Furthermore, these also suggest that AurA may acquire non-mitotic functions when deregulated in tumor cells or exposed to a tumor microenvironment.

Interestingly, the functional diversity of AurA in cancer makes it an attractive target in cancer. Moreover, AurA can be efficiently targeted by specific inhibitors. Indeed, AurA exhibits a specific ATP-binding pocket conformation, which can be recognized by specific inhibitors. Notably, Alisertib (MLN8237) is highly specific to AurA (> 200-fold than AurB), induces low to moderate adverse effects and shows encouraging results in phase II clinical trials for solid tumors, including breast and lung tumors [150, 170]. However, as AurA also plays a tumor suppressive role in stem cells, long-term secondary effects should be cautiously monitored. On the other hand, phase I clinical studies using GSK1070916A, a AurB/AurC inhibitor, has been completed in 2013 and not yet pursued into phase II [171]. It is conceivable that AurA specific inhibitors may be more efficient in counteracting tumor growth and dissemination with limited adverse effects.

## Abbreviations

ACD: asymmetric cell division
APC/C: anaphase promoting complex/cyclosome
aPKC: atypical Protein Kinase C
AIK: Aurora/IPL1-related kinase
ARK: Aurora-related kinase
AURK: Aurora kinase
Aur: Aurora
ARD1: Arrest Defective Protein 1
ATM: ataxia telangiectasia mutated
ATR: ATM and Rad3 Related
Bora: protein aurora borealis
BRCA1: breast cancer 1
CaM: Calmodulin
CSC: cancer stem cell
CDC25: dual specificity phosphatase Cdc25
CDE: cell cycle-dependent element
Cdh1: APC/C activator protein
CDK: cyclin-dependent kinase
Chk: checkpoint kinase
CHR: cell cycle gene homology region
CKAP: cytoskeleton-associated protein
Cnd2a: condensin complex subunit 2
CPC: chromosome passenger complex
CPE: cytoplasmic polyadenylation element
CPEB: cytoplasmic polyadenylation element binding protein
CTA: cancer testis antigen
d.p.c: day post coitum
D-box: degradation box
DCTN1: dynactin subunit 1
DSB: double strand breaks
ESC: embryonic stem cell
EMT: epithelial-to-mesenchymal transition
FAK: focal adhesion kinase
Fzr: fizzy-related protein homolog
Gadd45a: growth arrest and DNA damage-inducible protein GADD45 alpha
GVBD: germinal vesicle breakdown
HNSCC: head and neck squamous cell carcinoma
HP-1: heterochromatin protein 1
HR: homologous recombination
INCENP: inner centromere protein
Ipl1: increased in ploidy
Kif: kinesin family member
LATS2: large tumor suppressor 2
LIM: Lin11, Isl-1 and Mec-3
MT: microtubule
MAP: microtubule-associated protein
MEF: mouse embryonic fibroblasts
MI/II: Meiosis I/II
MKLP: mitotic kinesin-like protein
MMP: matrix metalloproteinase
MTOC: microtubules organizing center
NB: neuroblast
NDEL1: nuclear distribution protein nudE-like 1
NEBD: nuclear envelope breakdown
NHEJ: non-homologous end joining
NPM: nucleophosmin
PAR6: partitioning defective protein 6
PARP1: poly(ADP-Ribose) polymerase 1
PCM: pericentriolar material
PLK: polo-like kinase
PLZF: promyelocytic leukaemia zinc finger protein
PP1: protein phosphatase 1
RacGAP: Rac GTPase-activating protein
RASSF1A: Ras association domain-containing protein 1
SAC: spindle assembly checkpoint
SCFβ-TrCP: Skp, Cullin, F-box containing complex, β-transducin repeat-containing protein
Ser: serine
SGO1: shugoshin
SLiMs: short, linear motifs
TACC3: transforming acidic coiled–coil-containing protein 3
Thr: threonine
TPX2: targeting protein for Xenopus kinesin-like Protein 2
Tzfp: testis zinc finger protein
WT: wild-type
